# Data on expert system-econometric entropy informatics model for adjudicating residential building project costs

**DOI:** 10.1016/j.dib.2018.08.177

**Published:** 2018-09-11

**Authors:** Lekan M. Amusan, Ayo K. Charles, Ebunoluwa Adeyemi, Opeyemi Joshua, Ojelabi A. Raphael

**Affiliations:** Covenant University College of Science and Technology, PMB 1023, Canaanland Ota, Ogun State, Nigeria

**Keywords:** Questionnaire, Utility parameters, Likert scale, Cost entropy, Adjudication

## Abstract

This data article presents an expert system and econometric entropy-based informatics model for residential building project for cost judgment and decisions in residential building project. The data was obtained using purposive sampling technique to select projects completed between 2009 and 2011in Lagos state Nigeria, the project were examined for their cost centres. Also, As-built cost of one thousand(1000) samples of trained As-built cost of residential building projects trained with Neural network with Levenberg Marqua after being adjusted and modified with econometric factors like inflation index, cost entropy and entropy factor to stabilized the data and were used to form and train neural network used. Probability technique was used to generate risk impact matrix and influence of entropy on the cost centres. A parametric model similar to hedonic models was generated using the utility parameters within the early and late elemental dichotomy. The model was validated through comparative analysis of the econometric loading attributes using Monte Carlo technique of SPSS software extracting the contingency coefficient. The data of the model can provide solution to the problems of knowing the cost implication of a future project and also enable a builder or contactor load cost implication of an unseen circumstance even on occasion of deferred cost reimbursement.

**Specifications table**

**Table:** 

**Subject area**	*Building Construction; Construction Management.*
**More specific subject area**	*Artificial Intelligence Application*
**Type of data**	*Table, text file.*
**How data was acquired**	*Survey, Artificial Neural Network [Neuro Tools]*
**Data format**	*Raw.*
**Experimental factors**	**a. Data Training**: The training data set (1000 samples) of residential building projects having being modified with inflation index and exigency factor, was used to train the multilayered perceptron neural network selected, so as to select its parameters, the one suitable to problem at hand. Back propagation was used to train the network.
**Experimental features**	Back propagation was used to train the network since it is recommended and simple to code. So also gradient descent momentum and learning rate parameters was set at the start of the training cycle (for speed determination and network stability, range of momentum 0.1≤ *x ≤* 1, high = weight oscillation coefficient).
	It develops the input to output, by minimizing a mean square error (MSE) cost function measured over a set of training examples. The M.S.E. is given by this relation:
	*M.S.E = [(square root of [[[summation). Sub. (i=1). Sup.n) [(xi – E (i)].sup.2]]]*/*n*
	**b. The testing phase**: Data from remaining 1000 samples were used as testing data set to produce output for unseen sets of data. A spread sheet simulation program on Microsoft excel was used to test the generated model, according to optimized weights, comparison was made between actual cost and neural network cost, using cost percentage error (CPE) and mean estimated error (MEE).
	CPE = [[Enn – Bv]/[Bv]]×100%
	MEE = [1/*n*][[ I = n]. summation over (i = 1)] cpe(i)
**Data source location**	*The data was sourced from Construction Firms in Lagos state and Bureau of Statistics Abuja Nigeria, Nigeria.*
**Data accessibility**	*Data is with the article.*

**Value of the data**

● The data would be useful in assisting builders, engineers and all categories of site practitioners in using econometric models to determine magnitude of cost implications on construction sites.
● The data could enable client and tenderers to decide correctly on site cost issues.
● The data provides platform for further research in Application of Artificial Intelligence in solving construction problems.
● It provides basis for literary and practical contribution in the field of construction economics research.

## 1 Data

The data being presented includes: Factoring Elemental Cost Centers Influence on Project Cost, Entropy Level and Risk Threshold Perspective on Project Cost, Cost and Risk Impact Prediction Probability Matrix, Cost monetary Entropy Summary of Adjusted Projects B.O.Q Value and As-built Cost of 4-Bedroom Duplex, Cost Schedule for 2-Bedroom Bungalow, Early And Late Constructible Elements Monetary Entropy for Sampled Residential Buildings, Project Cost and Corresponding Neural Network Based-Entropy 2&3-Bedroom Bungalow, Structural Component of Neural Network Econometric Modified Back-End Loading Approach, Comparative Analysis of The Econometric Loading Attributes of Neural-Network Econometric Entropy-Based Model, Cost Limit Component Validations, Econometric Loading Attribute.

## 2 Experimental design, materials and methods

### 2.1 The training stage

The training data set One thousand (1000) samples of residential building projects were modified with inflation index and exigency factor. It was used to train the multilayered perceptron neural network with Levenber Marqua selected, so as to select its parameters, the one suitable to problem at hand [1], [2], [3], [4], [5], [6], [7]. Back propagation was used to train the network since it is recommended and simple to code. So also gradient descent momentum and learning rate parameters was set at the start of the training cycle (for speed determination and network stability, range of momentum 0.1≤ *x* ≤ 1, high = weight oscillation coefficient [7], [8], [9], [10].

It develops the input to output, by minimizing a mean square error (MSE) cost function measured over a set of training examples. The M.S.E. is given by this relation [11], [12], [13].

(1) M.S.E=[(squarerootof[[[summation).Sub.(i=1).Sup.n)[(xi–E(i)].sup.2]]]/n

### 2.2 The testing phase

Data from 1000 samples were used as testing data set to produce output for unseen sets of data. A spread sheet simulation program on Microsoft excel was used to test the generated model, according to optimized weights, comparison was made between actual cost and neural network cost, using cost percentage error (CPE) and mean estimated error (MEE).

(2) CPE=[[Enn–Bv]/[Bv]]×100%

(3) $$MEE=\left[\frac{1}{n}\right]\left[\left[I=n\right].summation\;over\left(i=1\right)\right]cpe\left(i\right)$$

The data presented in the table above is on ordinal scale of 0 to 1. The risk associated with project cost center can be quantified in term of degree of uncertainty,( probability of occurrence and magnitude of impact( i.e on project objective, quality and time). However, in simpler terms, a criterion value, ranking or status for each risk event (or set of combined events) may be established by dividing the frequency of relevant events by total number of possible events. In this section therefore. According to Amusan et al. [1], a planner should consider both financial assignment that will minimize project risk and maximize cost and also financial assignment that will maximize profit and prevent project disarray. Therefore at tender stage, elemental components with high risk factor should be considered first since they attract higher risk.

## 3 Entropy level and risk threshold perspective on project cost

The risk associated with project cost center can be quantified in term of degree of uncertainty, (probability of occurrence and magnitude of impact( i.e on project objective, quality and time).

### 3.1 IMPACT/CONSEQUENCE

The data presented in the table above is ordinal in nature. Data in Table 1.2 above contain comparative analysis of risk elements of 4–bedroom duplex, 2&3-bedroom Bungalow, 1-bedroom Apartment, 3 /4-bedroom, 24 Units 4 Floors risk elements with risk implication on the project. The following elements have high risk implications on Residential building Duplex and Bungalow: Substructure, Finishing, Frame, Service, Upper floor and Roof. More attention on those elements would help prevent financial wastage and in balancing of cost at tendering stage. Also the contents with high risk impact in Residential buildings with more floors include Frame and Finishing.

**Table 1.1:** Factoring elemental cost centres influence on project cost.

**S/N**	**Elements**	**Cost rating on scale probability (*P* = 0.0–1.0)**			
**C.**		4-Bedroom Duplex	2/3- Bedroom Bungalow	1- Bedroom Apartment	3&4- Bedroom, 4 Floors
**ELT1**	Substructure	1.0	1.0	1.0	0.8
**ELT2**	Frame & Walls	1.0	1.0	1.0	1.0
**ELT3**	Stair Cases	0.2	–	–	0.3
**ELT4**	Upper Floor	0.9	–	–	0.4
**ELT5**	Roofs	0.7	1.0	1.0	0.4
**ELT6**	Windows	0.5	0.4	0.5	0.5
**ELT7**	Doors	0.6	0.5	0.5	0.5
**ELT8**	Finishing	1.0	1.0	1.0	0.1
**ELT10**	Fittings	0.2	0.3	–	0.6
**ELT11**	Services	0.7	0.7	0.6	0.7
**ELT12**	Soil Drainage	0.2	0.2	0.7	0.6
**ELT13**	Preliminaries	0.4	0.4	0.5	0.7
**ELT14**	Contingencies	0.3	0.2	0.3	0.3
**ELT15**	Value Added Tax (5%)	0.5	0.5	0.5	0.1

**Table 1.2:** Data of probability matrix for predicting projects cost and risk impact [Probability Scale of 0.0–1.0].

#### 3.1.1 Evaluating project cost monetary entropy

Data of cost distribution pattern was presented in the analysis presented in Table 1.2, Table 1.3. The data presented is categorical in nature. It follows a pattern of law of inverse proportions. The lower the cost variation the lower the degree of probability variations produced, and consequently the lower the entropy and vice versa. The entropy mentioned here is the index used to quantify the degree of cost restiveness on the project. The movement could be traced to incessant price changes on account of macro and micro economic variables. The projects used in this work were executed during the economic meltdown period; this is adjudged as one of the factors that could lead to the price movement and disparity in cost-entropy obtained. The dynamic nature of price movement in a project being executed often dictates the pace of magnitude of the entropy.

**Table 1.3:** Summary of adjusted projects B.O.Q value and as-built cost of 4-bedroom duplex year 2006–2009.

**Cost Centers**		**1**	**2**	**3**	
	Project	A	B	C	
		B.O.Q Initial Value	As-Built Cost	Cost Variation	Percentage Entropy
**Project 1–11**	1	16,043,869	22,676,000	6632131	29
**Residential**	2	16,500,603	23,565,000	7064397	30
**Building**	3	16,225,501	24,113,000	7887499	33
**2009**	4	16,400,521	27,654,000	11253479	41
	5	17,100,438	22,221,000	5120562	23
	6	17,300,113	28,450,000	11149887	39
	7	16,800,073	30,500,000	13699927	45
	8	17,220,134	26,350,000	9129866	35
	9	16,210,687	25,800,120	9589433	37
	10	18,500,936	23,450,000	4949064	
	11	16,360,084	20,650,000	4289916	21

Data of projects is presented in Table 1.4 with percentage variation of 113 [percentage variation of Initial contract sum from As built contract sum of residential building projects while the least variation value was obtained at 36 percent of variation of As built building cost from Initial Tender value.

**Table 1.4:** Table cost schedule for 2-bedroom bungalow.

**Cost centers**		**1**	**2**	**3**	
	Project	A	B	C	
		B.O.Q Initial Valuet[Tender cost]	As-Built Cost	Cost Variation(B-A)	Percent Var
**Project 1-20**	1	3,085,100	4,236,000	1,150,900	36
**Residential**	2	3,171,800	5,800,000	2,628,200	83
**Building**	3	2,610,000	4,800,000	2,190,000	84
**2009**	4	3,165,000	4,350,000	1,185,000	37
	5	2,145,000	4,325,000	2,180,000	102
	6	3,174,953	4,286,350	1,111,397	35
	7	2,750,000	5,850,000	3,100,000	113
	8	2,700,850	5,121,000	2,420,150	90
	9	3,150,000	6,265,000	3,115,000	99
	10	2,766,000	5,223,000	2,457,000	89
	11	2,510,000	6,371,000	3,861,000	154

#### 3.1.2 Early and late constructible elements monetary entropy for sampled residential buildings

This data presented above can help in determining the rate at which cost of each elements could vary relative to elements of a project. The data indicated that Doors and finishing cost has the most frequent fluctuation, followed with frame and windows. The cost of those elements need to be properly taken into consideration in order not to delay work or affect entire project negatively. The cost entropy presented in the table could help contractors to achieve the purpose (Table 1.5).

**Table 1.5:** **Projects Particular 2&3‐Bedroom Bungalow**.

**S/N**	**Element**	**Tender Cost**	**Tagged Project Cost**	**Relative Percent**	**Relative Probability**	**Relative Entropy**
**B.**						
**ELT1**	Substructure	2,669,340	11,674,519.50	22.865	0.23	2.34
**ELT2**	Frame & Walls	1,519,415	11,674,519.50	13.015	0.08	2.49
**ELT3**	Roofs	1,197,000	11,674,519.50	10.253	0.10	2.47
**ELT4**	Windows	517,650	11,674,519.50	4.434	0.23	2.34
**ELT5**	Doors	544,500	11,674,519.50	4.664	0.05	2.52
**ELT6**	Finishing	2,541,535	11,674,519.50	21.770	0.05	2.52
**ELT7**	Fittings	298,800	11,674,519.50	2.560	0.39	2.18
**ELT8**	Services	786,350	11,674,519.50	6.736	0.15	2.42
**ELT10**	Soil Drainage	274,000	11,674,519.50	2.347	0.43	2.14
**ELT11**	Preliminaries	500,000	11,674,519.50	4.283	0.24	2.33
**ELT12**	Contingencies	270,000	11,674,519.50	2.313	0.43	2.14
**ELT13**	Value Added Tax (5%)	555,929.50	11,674,519.50	4.762	0.21	2.37

#### 3.1.3 Stabilizing cost centers for an optimum cost using neural network

The training data set (1000 samples) of residential building projects were selected, having being modified with inflation index and exigency factor, was used to train the multilayered perceptron neural network selected, so as to select its parameters, the one suitable to problem at hand. Back propagation was used to train the network since it is recommended and simple to code. So also gradient descent momentum and learning rate parameters was set at the start of the training cycle (for speed determination and network stability, range of momentum 0.1≤ *x ≤* 1, high = weight oscillation coefficient). The output is presented in Table 1.6 [9], [10], [11], [12], [13], [14].

**Table 1.6:** **Data on training of project cost of 2&3-bedroom bungalow with neural network**.

**Project**	**Tender cost(N)**	**Tagged cost(N)**	**Neural output(N)**	**Relative entropy**
**Prj 1**	3,085,100	4,236,000	7,3672,737	0.70
**Prj 2**	3,171,800	5,800,000	7,345,657	0.84
**Prj 3**	2,610,000	4,800,000	6,794,688	0.64
**Prj 4**	3,165,000	4,350,000	6,635,806	0.39
**Prj 5**	2,145,000	4,325,000	6,855,924	0.87
**Prj 6**	3,174,953	4,286,350	6,654,957	0.69
**Prj7**	2,750,000	5,850,000	6,592,822	0.67
**Prj8**	2,700,850	5,121,000	6,516,743	0.42
**Prj9**	3,150,000	6,265,000	6,872,945	0.50
**Prj10**	2766000	5,223,000	6,669,763	0.42
**Prj11**	2510000	6,371,000	6,587,965	0.61
**Prj12**	3268000	6,250,000	6,983,746	0.51
**Prj13**	2,250,325	5,675,000	6,857,236	0.42
**Prj14**	3,520,000	6,600,000	6,837,329	0.52
**P rj15**	2,100,000	5,125,000	6,787,856	0.43
**Prj15**	3,173,000	5,652,000	6,348,498	0.45
**Prj16**	3,173,000	7,650,000	6,575,585	0.44
**Prj17**	2,580,315	6,131,000	6,257,278	0.43
**Prj18**	2,420,500	5,643,000	6,468,567	0.44
**Prj19**	3,143,000	7,266,000	6,634,734	0.46

Data of selected Nineteen (19) project samples of the 1000 building projects sample. Nineteen(19 Neural network trained samples which are found to be consisted in value were selected and presented in the Table 1.6 above. The lowest cost indicated in the data above is for lowest cost generated through neural network training of the data trained by the neural network; the cost is N6,635,806 with corresponding cost entropy of 0.39.The highest cost entropy generated is 0.87 with cost of N6,855,929. The cost data range therefore that could be chosen as As-built cost of 2–3 bedroom bungalows. To predict future cost of construction cost,entropy presented could be factored into any cost to predict the future value. The data found utility in developing hedonic model such as presented in Section 3.1.5.

#### 3.1.4 Data of the expert system and econometric entropy-based model for residential building project cost adjudication

The expert system and econometric entropy-based model for residential building project cost adjudication is presented in this section. Three techniques were used to determine cost benchmark for each of the component of project elements. The early constructible element- loading, late-constructible element loading and individual- rate loading. This towed the line of submissions of [5] of front end loading, back-end loading and individual loading [13], [14], [15], [16].

#### 3.1.5 Data on structural equation of developed neural network econometric modified back-end loading model

A structural hedonic equation was previously developed, which could be used to generate data for adjudication of various project elements and problem of cost implication determination. The probability matrix of Table 1.1, Table 1.2, Table 1.3, Table 1.4 was used to generate data presented in Section 3.1.6.

$Developed\;Neural\;Network\;Econometric\;Modified\;Back-End\;Loading\;Model=Pjec=[\Sigma {\left(1-r\right)}^{-n}]([C\lambda_{nj}\left[\gamma_{nj}Exf–C^{1}\right)]+\lambda_{nj}[Q_{j}+Q_{i}][\gamma_{nj}Exf_{j}–C^{1})])$where rj --- Monthly Discount rate;

n --- Period in Consideration; C^1^--Actual Increase in Cost of Items; λ_nj --- Proportion of Elements;_
Q_j_; Q_i ---- Bill Cost of Item i, j;_ γ_nj --- Adjustment for Cost Escalation(risk factor);_ ----Exigency Factor(project entropy = 2.36) and C^1 ----^ unit cost of project element; Pjec – Project Element Cost.

#### 3.1.6 Data on validated developed neural network econometric modified back-end loading model using comparative analysis of the econometric loading attributes

Three techniques were used to determine cost benchmark for each of the component of project elements. The early constructible element- loading, late-constructible element loading and individual- rate loading. This towed the line of submissions of [13], [14], [15]. Brown and Rose [4]; Bajari and Benkard [2]; and Cattel, Bowen and Kaka [5] of front end loading, back-end loading and individual loading. Data of treatment of the project data with the three loading models mentioned was used to generate comparable project cost for tendering purpose and other purpose (Table 1.7).

**Table 1.7:** **Data on structural equation of developed neural network econometric modified back-end loading model using (2&3-Bedroom Bungalow)**.

**B.**	**Element**	**Tender cost**	**Tagged project cost**	**Front-end loading**	**Individual-rate loading**	**Data treated with developed structural equation**
						
**ELT1**	Substructure	2,669,340	11,674,519.50	3,012,567.00	737,298.40	2,939,503.9
**ELT2**	Frame & Walls	1,519,415	11,674,519.50	3,397,217.00	419,672.62	1,673,190.0
**ELT3**	Roofs	1,197,000	11,674,519.50	3,505,064.80	987,525.00	1,318,148.4
**ELT4**	Windows	517,650	11,674,519.50	3,735,654.40	142,980.11	570,041.41
**ELT5**	Doors	544,500	11,674,519.50	3,726,665.30	150,396.40	599,609.10
**ELT6**	Finishing	2,541,535	11,674,519.50	3,058,058.00	701,997.38	2,798,763.8
**ELT7**	Fittings	298,800	11,674,519.50	3,8018,925.70	82,531.60	329,041.60
**ELT8**	Services	786,350	11,674,519.50	312,645,694.0	217,198.00	865,936.80
**ELT10**	Soil Drainage	274,000	11,674,519.50	3,817,228.70	75,681.54	301,731.54
**ELT11**	Preliminaries	500,000	11,674,519.50	3,741,563.90	138,105.00	550,605.00
**ELT12**	Contingencies	270,000	11,674,519.50	3,818,567.90	74,576.7.0	297,326.70
**ELT13**	Value Added Tax (5%)	555,929.5	11,674,519.50	3,722,838.70	153,553.30	612,195.20

The data would be much useful for purpose of tender reparation. Due attention should be given to the Substructure and Finishing since they emerged as the elements with high cost of execution successful allocation would guarantee 80% success of the project. The cost category developed with the data treated with developed structural equation showed stability than other two, therefore recommended for use in project cost prediction.i.e. data treated with developed structural equation.

Data of Re-sampling test was presented on the model in order to ascertain the stability and the influence of outliers on the models’ stability. The data results are presented in Table 1.8, Table 1.9; two models are presented here, model of as-built sum and Econometric Front-end Loading and Individual-rate loading has standard error of 0.233. The two models can help in tender sum preparation to load cost implication of unseen variables that could help in tender sum prediction. The two models showed stability with high level of tolerance.

**Table 1.8:** **Cost limit component validations of the developed neural network econometric modified back-end loading model**.

**Elements and Statistical Parameters**		**4-bedroomduplex**	**2/3-bdrmbunglw**	**1-bdrm bung**	**3-bdrm,3-floors**
**4-bedrmdplx**	**Pearsons Corr.**	1.00	–	–	–
	**Sig.(2-tailed)**	0.00	–	–	–
					
**2/3-bedrmbung**	**Pearsons Corr.**	0.89	1.00	–	–
	**Sig.(2-Tailed)**	0.001	0.000	–	–
					
**1-bedrm bunglw**	**Pearsons Corr.**	0.886	0.895	1.000	–
	**Sig.(2-Tailed)**	0.001	0.000	0.000	–

**Table 1.9:** **Econometric loading attributes developed neural network econometric modified back-end loading model**.

Monte Carlo technique		Value	Asymp. Std. Error^b^	Approx. Sig.	Sig.	Lower boundary
99%	Confidence					
Interval						
Individual-rate Loading	Contingency-Coefficient	.957	.233	1.000	1.000^a^	1.000
	Kendall׳s tau-c	.909	.000	.000	.000^a^	.000
						
Econometric Front-end Loading	Contingency -Coefficient	.95	.233	1.000	1.000^a^	1.000
	Kendall׳s tau-c	1.00	–	.000	.000^a^	.000
						
Econometric Back-end Loading	Contingency -Coefficient	.967	.233	.233	1.000^a^	1.000
	Kendall׳s tau-c	1.00			.000^a^	.000
